# Game-Changing Approaches in Sperm Sex-Sorting: Microfluidics and Nanotechnology

**DOI:** 10.3390/ani11041182

**Published:** 2021-04-20

**Authors:** Andra-Sabina Neculai-Valeanu, Adina Mirela Ariton

**Affiliations:** 1 Research and Development Station for Cattle Breeding Dancu, 707252 Iasi, Romania; mirela.ariton@scdb-dancu.ro; 2 Department of Fundamental Sciences in Animal Husbandry, Faculty of Food and Animal Sciences, University of Applied Life Sciences and Environment “Ion Ionescu de la Brad”, 700490 Iasi, Romania

**Keywords:** sperm, sex-sorting, animal breeding, nanotechnology, microfluidics

## Abstract

**Simple Summary:**

Sexing of sperm cells, including the capacity to preselect the sex of offspring prior to reproduction, has been a major target of reproductive biotechnology for a very long time. The advances in molecular biology, biophysics, and computer science over the past few decades, as well as the groundbreaking new methods introduced by scientists, have contributed to some major breakthroughs in a variety of branches of medicine. In particular, assisted reproduction is one of the areas in which emerging technologies such as nanotechnology and microfluidics may enhance the fertility potential of samples of sex-sorted semen, thus improving the reproductive management of farm animals and conservation programs. In human medicine, embryo sex-selection using in vitro fertilization (IVF) and preimplantation genetic testing (PGT) is accepted only for medical reasons. Using sex-sorting before IVF would enable specialists to prevent sex-linked genetic diseases and prevent the discharge of embryos which are not suitable for transfer due to their sex.

**Abstract:**

The utilization of sex-sorted sperm for artificial insemination and in-vitro fertilization is considered a valuable tool for improving production efficiency and optimizing reproductive management in farm animals, subsequently ensuring sufficient food resource for the growing human population. Despite the fact that sperm sex-sorting is one of the most intense studied technologies and notable progress have been made in the past three decades to optimize it, the conception rates when using sex-sorted semen are still under expectations. Assisted reproduction programs may benefit from the use of emergent nano and microfluidic-based technologies. This article addresses the currently used methods for sperm sex-sorting, as well as the emerging ones, based on nanotechnology and microfluidics emphasizing on their practical and economic applicability.

## 1. Introduction

In the 20th century alone, the world’s population has grown from 1.65 billion to 6 billion and currently is estimated to be growing at a rate of around 1.08% per year—on average, 82 million people per year. According to the United Nations Food and Agriculture Organization, there will be 9.5 billion people by 2050, and this growth associated with economic development in several mid-income countries will implicitly bring changes in diet, so the demand for animal products will increase. However, feeding more than 9 billion people will not be an easy task, so the livestock sector will be under an extreme pressure for ensuring both quantity and quality of the products [1,2].

One of the most successful strategies for improving the genetic progress and profitability of cattle farms is the selection of offspring of a certain sex. Sperm-sorting, one of the most valuable instruments in assisted reproduction, allows for the selection of “healthy” sperm, as well as the determination of specific traits, such as sex, process in which sperm cells are separated into two distinct populations with X-bearing (female), respectively Y-bearing (male) chromosomes [3,4,5]. The utilization sex-sorted sperm for artificial insemination and in vitro fertilization is considered a valuable tool for improving production efficiency and optimizing reproductive management in farm animals, subsequently ensuring sufficient food resource for the growing human population [6,7,8].

This article addresses the currently used methods for sperm sex-sorting, as well as the emerging ones, emphasizing their practical and economic applicability.

## 2. Overview of the Sex-Sorting Importance

The preselection of semen based on the sex chromosome is a commonly used biotechnology in the animal livestock industry for obtaining offspring of a desired sex; it is important for improving animal breeding efficiency and providing sustainable milk and beef production for the human population, being well accounted for by the Food and Agriculture Organization (FAO). The utilization of sex-sorted semen is becoming more and more popular all around the world, among farmers, mainly due to the numerous advantages it may bring. Besides extending the size of herds faster, in a more cost-effective manner, potential bio-security threats may be avoided [9,10,11].

Globally, the cattle-breeding industry has benefited from the use of this technology in both intensive and pasture-based systems [12,13,14,15,16]. For example, in dairy cows, female calves are essential to increase productivity, while male calves have a lower economic value. The higher value of female calves and the reduced risk of dystocia are two other powerful economic factors for reducing the number of male calves raised in the dairy industry. According to a study conducted by Seidel (2003) [17], dystocia may be reduced to up 20% when sex-sorted semen is used for artificial insemination (AI). Another study carried out by Norman et al. (2010) [18] found that using sexed semen reduced dystocia risks in heifers by 28%, respectively by 64% in cows. This finding may be due to the fact that a heifer, on average, is 2 kg lighter in weight at birth as compared to male calves [17]. The male-to-female ratio is really not precisely 1:1, since many variables, including nutrition, may alter it. Garner and Seidel (2008) [19] showed that the usual sex ratio for artificial insemination is around 51% male, with older cows producing 53% male offspring. Therefore, specific and efficient reproductive technology for sperm sex-sorting would be immensely helpful for the dairy sector. In contrast, in beef farms, male calves are those whose economic value makes a difference [20,21]. There is also interest in other species, such as equines, as well, for the use of sex-sorting semen [22]. Sex-sorted semen may be used to ensure rare equine breeds survive or for commercial purposes, especially in those breeds where a particular sex is required [23].

In horse polo and the Arabian Horse Show, females are favored over males because they are considered to be more agile and easier to train. In the swine industry, for example, female piglets may be preferred, since, this way, the costs associated with male castration are reduced [24]. The routine use of sex-sorted sperm in AI-programs will provide the swine industry with a whole new dimension.

Other emergent livestock industries, such as the donkey-milk industry, may benefit from this biotechnology in order to meet the increased market demand for jenny milk. Donkey milk has long been used as a substitute for children and patients with cow’s milk protein allergy, owing to its resemblance to human milk and low allergenic potential. In contrast to other types of milk conventionally used for human feeding, donkey milk has some superior biochemical and nutraceutical qualities [25,26]

This biotechnology may bring a genetic and economic boost in the sheep and goat industry, too [27]. Furthermore, sex-sorted semen has proven to be of great help in the programs designed for endangered species by improving the quality of the genetic bank resources (semen and embryos) [6,28,29,30,31].

In the case of animals, this biotechnology was implemented for the economic and genetic benefits it may bring, but the use of sex-sorting semen in human-assisted reproduction raised, as foreseen, many ethical controversies, which is why the use of sex-sorting technologies for family-balancing is prohibited in many countries [32,33,34]. For medical reasons, such as the prevention of sex-linked genetic disorders, embryo sex-selection using in vitro fertilization (IVF) and preimplantation genetic testing (PGT) is accepted. However, if the sperm is not sex-sorted prior to IVF, there is a chance of not obtaining embryos suitable for transfer, and the ones that cannot be transfer must be discharged. Thus, sex-sorting combined with IVF and PGT may increase the chances of obtaining a healthy offspring and even reduce the costs associated with IVF and PGT.

Despite the fact that sperm sex-sorting is one of the most intense studied technologies and notable progress have been made in the past three decades to optimize it, the conception rates when using sex-sorted semen are still under expectations [35,36]. Assisted-reproduction programs may benefit from the use of emergent technologies, such as nanotechnologies and microfluidics.

## 3. Conventional Approach—Flow Cytometry Sorting

Over the years, several different methods for sperm separation have been described, based on the various differences between Y- and X-bearing sperm, such as shape and size, ration, motility pattern, and surface charge. The reliability of some of the approaches has been doubted, primarily due to poor reproducibility and precision. One such example is the multiple gradient centrifugation, a method routinely used in human and animal assisted reproduction for the selection of sperm populations with specific characteristics, such as high chromatin integrity and improved motility and viability [37,38], as well as retrieval of spermatozoa form semen ejaculates [39,40,41]. Several authors used this method in combination with the swim-up technique for the separation of X- and Y-bearing sperm populations in different species, but the results were rather contradicting. The approach is based on the fact that during gradient centrifugation, the X-chromosome carrying spermatozoa will settle at the bottom of the column, due to the higher density of these cells and the Y chromosome bearing spermatozoa remains in high proportion at the top of the column. While some authors considered the multiple gradient centrifugation, using colloids such as Percoll, Bovipure or PureSperm, a useful tool for the enrichment of semen with X-bearing spermatozoa [42,43,44,45,46], others concluded that, although this method may slightly enrich X- or Y-bearing spermatozoa, the differences are insignificant and, implicitly, the method cannot be routinely used for sex-sorting semen [42,47,48,49,50,51].

Presently, the only effective sperm-sorting system is flow cytometry, a high-speed analysis tool for the counting and sorting of cells. This technology is commonly used in the medical field and clinical laboratories to perform for a large variety of assays [20,52]. For semen to be sorted using flow cytometry, firstly the sample must be diluted and labeled with a specific fluorescent dye called Hoechst 33342 which binds to the DNA of each sperm cell [3]. The X and Y chromosomes, also known as the sex chromosomes, differ significantly when it comes to DNA content, X-spermatozoa containing, on average, 3% more DNA in human and almost 4% in other livestock species, such as bulls, boars, rams, and rabbits, respectively [3]. Due to this difference, the X-chromosome-bearing spermatozoa will absorb a greater amount of dye. Subsequently, cells are then compelled to flow in single droplets into a laser contact region, to be irradiated by intense laser beams. Based on the exhibited fluorescence, the cell population may be calculated and the form and size of each cell may be assessed [53]. This basic approach has already been applied to sperm sex-sorting, especially in the dairy and beef sector, with several million calves being born from sex-sorted sperm. However, various studies have shown that the exposure of cells to certain wavelengths of the UV light spectrum, as well as the combination of fluorescent dye bis-benzimide (Hoechst 33342) used for cell labeling [4], may affect both sperm motility and membrane integrity [54,55,56,57,58].

**Figure 1:**
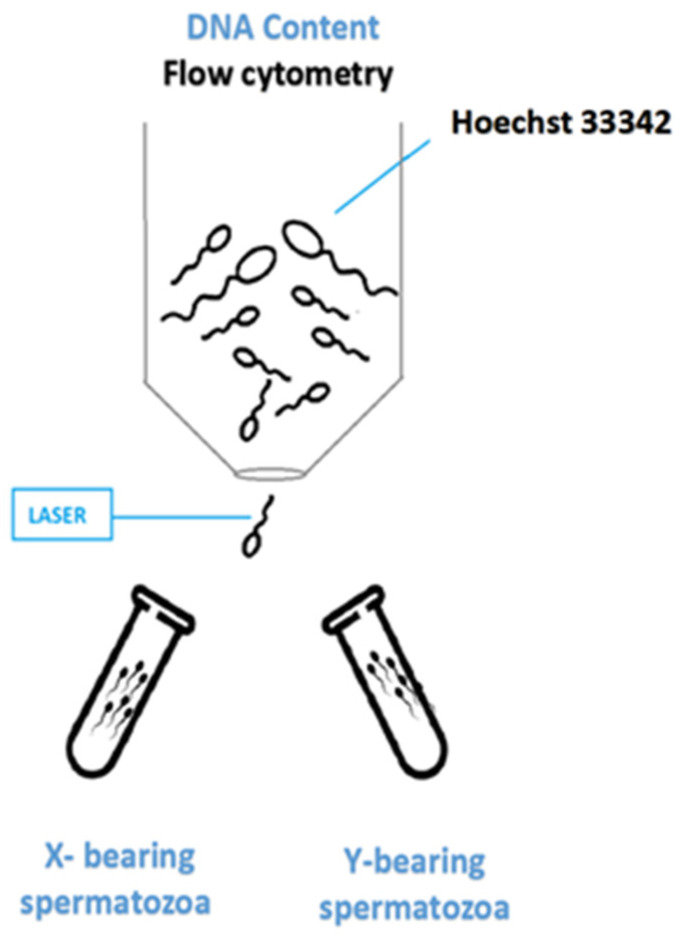
Schematic representation of conventional sex-sorting technique (Flow cytometry).

The post-separation purity of the separated sperm cells is then confirmed by in situ hybridization. The mechanical stresses induced on sperm cells during sorting and centrifugation increase the number of dead or damaged sperm cells by almost 20% [59]. Moreover, during the sorting process, there are several other stress factors that may intervene, such as shear forces acting during the hydrodynamic focusing and passage through the injection nozzle, repeated electrical doping corresponding to sperm DNA content, and the subsequent passage through the electrostatic deflection field. Apart from the sorting process, the steps that precede or succeed, such as co-incubation with the fluorescent dye, and chilling and storage at low temperatures before insemination, respectively, are responsible for further effects on sperm quality (Figure 1).

Sex-sorted semen is usually subject to a freezing and then thawing process; thus, its fertilizing ability is significantly reduced as compared to unsorted sperm, and it has constituted a major contribution in limiting the routine use of this method in the livestock industry [60]. During sex-sorting, the sperm cells must be individually evaluated; therefore, the concentration of sperm per dose is much lower, i.e., about two million sperms per dose compared to the unsorted semen doses, which usually have a concentration of about 20 million sperm cells [61]. More recent studies focused on increasing the sperm concentration of sex-sorting semen doses in an attempt to improve fertility, but this is more likely to increase the price per dose as well.

Despite more than 30 years of development, the fertilizing ability of sex-sorted spermatozoa is still behind farmers’ expectations; however, it is worth mentioning that the sorting speed has been improved significantly [62,63,64]. A study conducted by Steel et al. (2020) [65] concluded that the flow-cytometry sex-sorting alters sperm morphokinetics in a way that extends after fertilization, thus reinforcing the fact that sperm quality plays a vital role in the early development of embryos. These findings are also supported by the study led by Mostek et al. (2020) [66], according to which the enzymes involved in glycolysis, oxidative phosphorylation, and preservation of a steady energy charge were altered in sex-sorted semen as compared to non-sorted. In addition, the proteins which are involved in capacitation, acrosome reaction, and subsequently sperm–egg fusion are less abundant in sex-sorted semen, which could explain the lower fertility potential. In research conducted by Magata et al. (2021) [67], the developmental kinetics and viability of bovine embryos produced in vitro bovine by using sex-sorted semen was continuously monitored via time-lapse, and the authors showed that both embryo development and viability were impaired when using sex-sorted semen. Apart from the biological challenges, the fairly high cost of the equipment and the necessity of well-specialized personnel has led to the development of innovative, less expensive approaches for sperm selection and sex-sorting; thereby, biomarker-based nanotechnology and microfluidics became a topic of great interest.

## 4. Conventional and Sex-Sorted Semen Market—Present and Future Trends

The constant growing human population is one of the most significant factors driving the need for increasing animal productivity. Nevertheless, one of our most critical priorities in the coming decades would be not only to feed the planet but also to do it in a sustainable manner. For many people all over the world, meat and dairy are important sources of nutrition. For instance, meat production has more than tripled in the past 50 years; every year, almost 330 million tons are produced, of which 30% is beef. Global demand for meat is still increasing; however, according to FAO estimates, this increase will tend to slow down [68]. Simultaneously, many consumers are likely to expand their meat selection by including pricier meat proteins, such as beef and sheep meat, resulting in increases in global per capita consumption of these meat types by 2030 [69].

Demand for dairy products, especially cheese, is expected to increase as well in the coming years, resulting in higher EU milk production in the years ahead. However, meat and dairy production has significant environmental consequences, such as increased greenhouse gas emissions, agricultural land usage, and freshwater use. Producing and consuming meat, dairy, and other protein products in an environmentally friendly manner is one of the world’s most pressing challenges. To meet the EU’s sustainability goals of reducing greenhouse gas emissions, milk production will rise moderately to almost 180 million tons by 2030. Farming practices will most likely change, with an emphasis on herd management and nutrition [68].

Reproductive biotechnologies, such as artificial insemination, are one of the key components of an efficient farm management program. By 2028, the global demand for veterinary artificial insemination is projected to hit over USD 6 billion, rising at a compound annual growth rate (CAGR) of 5.94 percent between 2021 and 2028. This increase is generated by the requirement for genetically superior animal breeds, which may allow farmers to obtain higher yields from a lower number of animals, thus reducing the environmental impact as well. Associated benefits, such as the efficient use of reproductive males, genetic selection, reduced housing costs, and reduced risk of disease transmission, are expected to increase the adoption of reproductive biotechnologies in the coming years [70]. Moreover, the use of assisted reproduction biotechnologies for the conservation of endangered species may be propelling the sector even forward.

In either beef or dairy cattle, selecting the sex of the offspring may be one of the deciding factors in increasing genetic advancement and farmer profitability, since certain productive traits are closely related to gender. Using sex-sorted semen for artificial insemination of in vitro fertilization enables livestock producers to preserve or increase their herds without having to purchase additional females or exposing the herd to potential pathogens and diseases [71,72,73]. Additionally, the use of sex-sorted semen in conjunction with other reproductive biotechnologies has the potential of improving the global livestock management by allowing for the predetermination of an animal’s sex, thus maximizing the profitability of farms [74,75,76,77,78].

Notwithstanding the improvements made over the past years in sperm sex-sorting using flow cytometry, some authors concluded that, in both beef and dairy cattle, the conception rates are lower in comparison to unsorted semen [79,80,81]. For instance, in a field trial carried out by Borchersen and Peacock [82] on three different breeds (Holstein, Jersey, and Danish Red), a variable decrease in conception rate when using sex-sorted semen was observed, ranging from 12 percent in Holstein and 5 percent in Danish Red. Another study, performed by Seidel and Schenk (2008) [83], showed lower pregnancy rates from using sex-sorted semen as compared to non-sorted. The abovementioned findings are corroborated with more recent research carried out by Mikkopla et al. (2015), [84], Joezy-Shekalgorabi et al. (2017) [85], Dawod and Elbaz (2020) [81], and Drake et al. (2020) [86] regarding the reproductive efficiency of sex-sorted sperm in cows and heifers. However, when determining whether or not to use sex-sorted semen in a dairy farm, the climatic and management practices of a herd in a particular environment must be taken into account.

Despite the literature’s varying findings regarding conception rates in cows when sex-sorted semen is used, the majority of studies with heifers shows that the conception rate after AI with sex-sorted sperm ranges between 70% and 90%, depending on the farms management [61]. For these reasons, several recommendations were made for sex-sorted semen to be used in heifers in order to achieve better results.

In the human-assisted reproduction sector, the sperm bank market is expected to expand significantly in the coming years, as well. In 2018, the global sperm bank market was valued at USD 4.33 billion, and it is expected to grow at a CAGR of 3.3 percent between 2018 and 2025 [87]. There are several factors driving the growth of this sector, such as the advancements in cryopreservation techniques, increased awareness and access to infertility care, and the fertility tourism phenomena. Another major factor that may influence the market growth is the prevalence of obesity around the world, a disorder well recognized as one of the major risk factors for male and female infertility. The increasing acceptance of single-parent or same-sex families in many countries will also contribute to the potential growth opportunities of this industry.

The sperm preparation and analysis segment is expected to increase as the prevalence of infertility in the general population continues to increase. In developed countries, the sperm analysis market is expected to expand in response to rising demand for effective treatments and government policies to support infertility treatment. The growing popularity of cross-border reproductive care in countries like Spain, Czech Republic, or Ukraine, owing to the less restrictive legal frameworks and lower costs [88], has boosted demand for semen analysis services because comprehensive semen analysis is mandatory before any assisted-reproductive technology (ART) treatment.

In order to maintain their market share, industry players are constantly developing new products, as well as forming alliances and collaborations to ensure conventional and sex-sorted semen with improved fertility and better costs. Therefore, the increased demand for sexed semen and the emergence of new and improved sexing technologies is more likely to fuel the growth of the sexed-semen segment.

## 5. New Generation Technologies for Sperm Sex-Sorting: Microfluidics and Nanotechnology

Over the years, countless efforts have been made in establishing methods to separating X- and Y-bearing sperm populations and thereby obtaining offspring of desired sex in farm animals, for commercial purposes, or for human medical reasons (e.g., genetic sex-linked disorders). The international animal breeding industry is presently confronted with an unmet market need for an inexpensive and efficient sperm sex-sorting tool. Thereby, the development of alternative laboratory techniques for sperm sex-sorting has become an essential objective for scientists activating in the field.

Microfluidic platforms integrating electromanipulation technologies such as dielectrophoresis (DEP) are now widely used for cell analysis, biomedical applications, and environmental monitoring. With countless demonstrations of its ability to sort cells by using non-uniform electric fields, without the need for chemical labelling, the field of dielectrophoretic (DEP) has come again into the spotlight due to its potential to manipulate microparticles, nanoparticles, and cells. New applications are being discovered, as DEP separation is now transferred from laboratory settings to practical on-chip devices. This technique was previously successfully used on microfluidic chips for diagnosis purposes, such as the separation of circulating cancerous cells [89,90] and red blood cells [91]. In human and animal reproductive medicine, nanotechnology, microfluidics, and dielectrophoresis are now being used as useful tools for improving sperm quality, enabling the sorting of a sperm population with intact sperm membrane, high DNA integrity, and improved motility [92,93,94,95,96]. Assisted wildlife reproduction may also benefit from the latest breakthroughs and new horizons provided by these emergent technologies. Destructive human activities and climate change have led to habitat loss; thereby, extinction of animal species is currently occurring at a much faster rate than anticipated [97]. Conservation breeding and ARTs are precious assets in the struggle to save endangered wild animal species. In the near future, innovative approaches, such as microfluidic-based in vitro culture or IVF-on-a-chip, may be integrated into conservation programs and crucially contribute to the conservation of endangered species [98].

**Figure 2:**
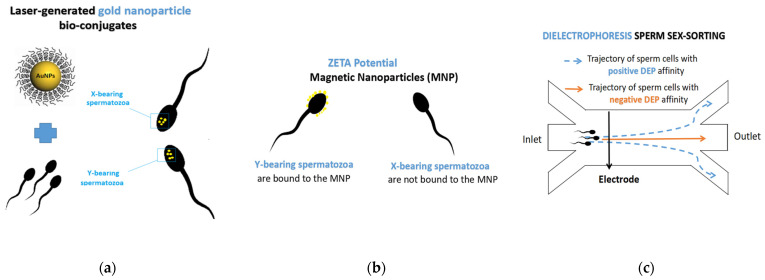
Schematic representation of emergent sex-sorting technologies: (**a**) laser-generated gold nanoparticles bio-conjugates, (**b**) Zeta potential, and (**c**) dielectrophoresis sperm sex-sorting.

Apart from sorting sperm cells based on certain characteristic, some studies focused, for example, on the qualitative identification of sex-related differences in sperm, using gold nanoparticles (AuNPs) [99,100]. One such study was conducted by Mancini (2015) [101], who proposed a novel approach for sperm gene targeting, based on vital genetic sequences detection, using laser-generated gold nanoparticle bio-conjugates (Figure 2).

According to the author, the research carried out concluded that the bovine Y chromosome has been shown to have an amount of triplex target sites, rendering triplex hybridization a valid option for the identification of Y-chromosome-bearing sperm. Another group of researchers focused on using magnetic nanoparticles for the sex-sorting of donkey semen. Nanoparticles (NP) have been used previously to separate apoptotic spermatozoa from the semen sample together with fluorescent dyes, antibodies, or magnetism, but it appears that this this technique may also be very successful in isolating X-bearing sperm cells without impacting the physiology and morphology of the spermatozoa. The magnetic nanoparticle (MNP) method is also based on the Zeta potential difference.

In brief, semen is combined with nano-size magnetic microbeads (approximately ~50 nm in diameter) which are negatively charged, incubated for 10 min, and then subjected for about 20 min to a magnetic field, for the selective isolation of X- or Y-bearing sperm (Figure 1). According to Domínguez et al. (2018) [102], Y-bearing spermatozoa exhibited a Zeta potential of −16 mV, while the X-bearing spermatozoa presented a Zeta potential of −20 mV; therefore, the Y-bearing sperm population will form complexes by binding more easily to the MNP. By applying a magnetic force to the test tube, the complexes will adhere to the inner wall of the test tube, while the X-bearing population remains suspended in the media and may be retrieved by slow aspiration (Figure 2). In donkey semen, sperm cells carrying the X chromosome were isolated, based on their Zeta potential, with an efficiency of 90%, using a magnetic field, without altering the functional biomarkers of semen, such as viability, motility, and chromatin integrity structure [102]. Preliminary experiments with magnetic nanoparticles, carried out on equines, showed that the pregnancy outcome when using magnetic sex-sorted stallion semen was nearly 80%, with 95% of conception products being confirmed as females, by ultrasound [103].

Recently, Wongtawan et al. (2020) [104] proposed a novel approach for sperm sex-sorting. The difference in membrane charge (Zeta potential) between X- and Y-bearing sperm cells may be exploited to separate the two populations, using a microfluidic dielectrophoretic-based chip to safely sex-sort sperm cells without affecting motility and viability (Figure 2). During electrophoresis, sperm cells reacted differently: The X-bearing population passed along the chip (negative DEP or nDEP), away from the electrode, while the Y-bearing population, referred to as positive DEP (pDEP), was drawn towards the electrode. The authors concluded that specific conditions such as 4 V voltage and 1 MHz frequency greatly decreased the percentage of Y-bearing sperm population to almost 30%, thereby enriching the X-bearing sperm population. However, various parameters, such as the type of buffer, flow rate, voltage, and frequency, influenced the sorting performance; thereby, further studies are needed to establish the most appropriate conditions and type of buffer that must be used to improve sex-sorting accuracy. 

Notwithstanding the effectiveness of flow cytometry in selecting subpopulations of cells using fluorescence markers, microfluidic-based sorting appears to be more a more suitable in approach for the selection of sensitive cells, such as cancer, sperm, blood, and immune cells [105]. Microfluidics enables sperm cells to be sorted in a simpler, time-effective, and gentler manner that more closely resembles natural selection processes while avoiding some of the more harmful aspects of conventional sperm-sorting techniques [106]. Unlike multiple gradient centrifugation, a time-consuming technique that requires highly skilled personnel, or flow cytometry, which requires costly equipment and fluorochromes, microfluidic sorting uses disposable chips that can be handled easily in any laboratory.

## 6. Conclusions

The flow cytometry sex-sorting method is detrimental to the sperm cells, especially affecting their viability. The urgent need to find new and improved methods for sex-sorting, along with the advances in micro- and nanofluidics, has led to the idea of using microfluidic flows and nanotechnology to separate sperm cells. Assisted-reproduction programs may benefit from the use of emergent sexing technologies that do not require expensive equipment and highly specialized human resources. Further studies should focus on the particularities of each species, as well as on the specific characteristic of individuals from the same species, in order to improve the accuracy of this method and convert it into a commercially available product.

## Data Availability

Not applicable.
